# Native knee kinematics is not reproduced after sensor guided cruciates substituting total knee arthroplasty

**DOI:** 10.1186/s40634-023-00567-2

**Published:** 2023-02-14

**Authors:** Pier Francesco Indelli, Michele Giuntoli, Karlos Zepeda, Stefano Ghirardelli, Rosa Susanna Valtanen, Ferdinando Iannotti

**Affiliations:** 1 Department of Orthopaedic Surgery, Stanford University School of Medicine, Stanford and the Palo Alto Veterans Affairs Health Care System (PAVAHCS), Palo Alto, USA; 2 Department of Orthopaedic Surgery, Stanford University School of Medicine, 450 Broadway, Redwood City, CA 94063 USA; 3 Department of Orthopaedic and Trauma Surgery, University of Pisa, Pisa, Italy; 4 Touro College of Osteopathic Medicine, New York, USA; 5 Südtiroler Sanitätsbetrieb, Bressanone, Italy; 6 Department of Orthopaedic and Trauma Surgery, San Paolo Hospital, Civitavecchia, Italy

**Keywords:** TKA, Gait, Total knee arthroplasty, Posterior stabilized, PS, Knee, Kinematic, Biomechanics

## Abstract

**Purpose:**

Gait analysis was used to evaluate knee kinematics in patients who underwent successful primary total knee arthroplasty (TKA) using two modern bi-cruciate substituting designs. The knee joint was balanced intraoperatively using real-time sensor technology, developed to provide dynamic feedback regarding stability and tibiofemoral load. The authors hypothesized that major differences exist in gait parameters between healthy controls and post-TKA patients.

**Methods:**

Ten patients who underwent successful TKA using bi-cruciate substituting designs were evaluated at a minimum of 9 months postoperatively using three-dimensional knee kinematic analysis; a multi-camera optoelectronic system and a force platform were used. Sensor-extracted kinematic data included knee flexion angle at heel-strike (KFH), peak midstance knee flexion angle (MSKFA), maximum and minimum knee adduction angle (KAA) and knee rotational angle at heel-strike. Multiple gait analysis data from the study group were compared to a group of ten healthy controls who were matched by age, sex and BMI. Clinical outcome in the TKA group was also measured using the Knee injury and Osteoarthritis Outcome Score (KOOS).

**Results:**

Clinically, at final follow-up, a statistically significant difference in pain, general symptoms, and activities of daily living was seen between the groups. From a gait analysis standpoint, TKA patients had significantly less rotation at heel strike (*p* = 0.04), lower late stance peak extension moments (*p* = 0.02), and less Knee Adduction Angle excursion during swing phase (*p* = 0.04) compared to the control group. No statistically significant difference was observed for knee flexion angle at heel strike, knee adduction moment, or peak knee flexion moment between the groups.

**Conclusions:**

Modern bi-cruciate substituting TKA designs failed to reproduce normal knee kinematics. The lack of full knee extension during the stance phase, absence of the “screw-home mechanism” typical of an ACL functioning knee, and the reduced fluctuation in knee adduction angle during the swing phase still represent major proprioceptive and muscular recruitment differences between normal and replaced knees.

## Background

Total Knee Arthroplasty (TKA) remains a very successful surgical treatment for knee arthritis. Unfortunately, 20% of post-operative patients still report major limitations in activities of daily living (ADLs) [40] compared to their age-matched non-arthritic peers [42]. Subjective symptoms (e.g. instability, abnormal proprioception) and objective clinical findings [e.g. poor range of motion (ROM), chronic joint effusions, inability to use the stairs, inability to comfortably kneel or squat] are commonly reported elements of patient dissatisfaction.

In an effort to improve patient satisfaction, the design of modern TKA underwent major modifications by many orthopaedic medical device companies over the last 10–15 years. The aim of this “implant personalization” was to update geometry and conformity [22] to restore normal knee kinematics; it followed the theoretical dogma that reproducing normal anatomy would provide a more natural joint proprioception. To make further progress towards this goal, the industry has also introduced digitalized technologies such as robotics, computer navigation, and load−sensing intra−articular sensors. The true impact of these technologies on knee kinematics during ambulation is yet to be proven. Previous studies from the current authors’ Institution supported the evidence that strong differences in knee kinematic behavior exist when tested in the stance phase of gait (joint center of rotation is on the lateral side) [28] compared to the swing phases of gait, stair climbing, and mini−squatting activities (joint center of rotation is on the medial side) [27, 44]. Reproduction of the “dual pivoting” knee kinematics [38] is extremely challenging with arthroplasty because the anterior and posterior cruciate ligaments (ACL, PCL)—which are commonly removed during the procedure—play a major role in normal knee kinematics [2, 10, 20, 31].

The aim of this study was to register knee kinematic parameters during normal gait (knee flexion–extension angle, adduction–abduction angle, internal–external tibial rotation, peak knee flexion moment, first peak knee adduction moment, and peak knee internal rotation moment) in patients who underwent successful bi−cruciate substituting TKA. Pressure sensors were used intra−operatively to obtain the desired articular stability. The acquired kinematic data was compared to match−paired healthy controls. The authors hypothesized that the kinematic parameters of TKA patients differ significantly from healthy controls because the proprioceptive role of the ACL and PCL is not replicated by modern posterior−stabilized TKA designs or by using a computer−assisted intraoperative strategy.

## Materials and methods

This was a retrospective case–control study. Patients who underwent primary TKA because of severe unilateral knee osteoarthritis at two authors’ Institutions (PFI, MG) were included in this study. Inclusion criteria consisted of age greater than 40 years with clinically and radiographically diagnosed unilateral tricompartmental osteoarthritis. Preoperative exclusion criteria included pre-existing concurrent hip, ankle, and/or contralateral knee osteoarthritis, presence of a chronic inflammatory disease, body mass index (BMI) greater than 35 kg/m^2^, and/or prior joint replacement surgery. The treatment group was composed of ten patients who underwent a bi-cruciate substituting TKA; five patients had a Persona posterior-stabilized (PS) implant (Persona, Zimmer-Biomet, USA) and five patients had a Legion posterior-stabilized (PS) TKA design (Smith & Nephew, London, UK).

The authors decided to use a second-generation PS design (Legion, Smith & Nephew, London, UK) and a third-generation (Persona, Zimmer-Biomet, USA) in order to highlight any difference in the gait data as well as in the clinical outcome. Intraoperative joint balancing was obtained using the load pressure system VERASENSE™ (Orthosensor Inc., Dania Beach, FL, USA).

The load sensor utilized in this study consists of a digital trial insert that can detect intra-operative tibio-femoral loads during component trialing. This technology can quantify the intercompartmental pressure and is able to report data on a screen, delivering a real-time feedback to the surgical team. In addition, the wireless sensor can define the tibio-femoral contact points during passive range of motion testing, including measuring the femoral roll-back during high flexion. Intraoperative load measurements were systematically taken at 10°, 45°, and 90° of knee flexion [33]. The current authors considered the knee well-balanced when the medial compartment pressure was 50 ± 20 pounds, the lateral compartment pressure was 35 ± 20 pounds, and the intercompartmental difference was within 15 pounds. An identical surgical technique was used in all ten cases: this was a combination of gap-balancing in extension and measured resection in flexion with uniform removal of both the ACL and PCL. All patients followed an identical postoperative rehabilitation protocol that included weight-bearing as tolerated with crutch-assist on the first postoperative day.

All patients were followed clinically at the same time intervals: 3-months, 6-months, and 9-months postoperatively: this final timeframe was selected since it has been reported that the kinematic of the knee following TKA has plateaued at nine months from the surgery [27].

At 9 months of minimum follow-up (FU), the treatment group was matched by gender, age, BMI and operating surgeon, to 10 healthy controls. The major postoperative inclusion criteria in the TKA group was the demonstration of a high Knee Injury and Osteoarthritis Outcome Score (KOOS) at final FU [34].

Patients in both groups were matched by age (TKAs: 67.8 ± 6.8 years; Control Group 59.4 ± 7.9 years), sex (all males) and BMI (TKAs: 32.8 ± 5.9 kg/m^2^_;_ Control group: 30.3 ± 4.6 kg/m^2^) (Table 1). All patients had to demonstrate full knee extension and at least 125° of active flexion prior to the gait analysis test.

**Table 1:** Study population demographics

	TKA	HEALTHY CONTROLS	*p*-VALUE
Age (years)	67.8 ± 6.8	59.4 ± 7.9	N.S
Sex	10 males	10 males	N.S
BMI (kg/m^2^)	32.8 ± 5.9	30.3 ± 4.6	N.S
KOOS pain score	75.0 ± 23.1	98.9 ± 1.9	< 0.01
KOOS symptoms score	71.4 ± 21.9	97.1 ± 4.1	< 0.01
KOOS ADL	81.2 ± 18.2	99.7 ± 0.9	< 0.01
KOOS Sports	60.0 ± 31.8	99.0 ± 2.1	< 0.01
KOOS QOL score	63.1 ± 31.0	96.9 ± 5.3	< 0.01
Forgotten Joint Score	50.2 ± 38.1	N/A	N/A
KFH (°)	5.0 ± 4.1	4.5 ± 3.6	N.S
Midstance KFA (°)	16.1 ± 5.2	20.7 ± 5.5	0.07 (trend)
Tibial rotation at heelstrike	5.8 ± 5.4	11.2 ± 5.5	0.04
Peak KAA during swing (°)	0.2 ± 4.2	-5.1 ± 5.7	0.04
KAA excursion during swing (°)	6.0 ± 2.3	9.4 ± 4.9	0.07 (trend)
KAM 1 (%BW^*^Ht)	2.16 ± 0.52	2.38 ± 0.67	N.S
Peak KFM (%BW^*^Ht)	2.91 ± 1.41	3.27 ± 1.08	N.S
Peak KIRM (%BW^*^Ht)	0.78 ± 0.17	0.77 ± 0.27	N.S

*TKA* Total Knee Arthroplasty group, *BMI* Body Max Index, *WOMAC* Western Ontario and McMaster Universities Arthritis Index, *KOOS* Knee injury and Osteoarthritis Outcome Score, *KFH* Knee Flexion angle at Heel-strike, *KFA* Knee Flexion Angle, *KAA* Knee Adduction Angle, *KAM* Knee Adduction Moment, *KFM* Peak Knee Flexion Moment, *KIRM* Knee Rotational Moment, *N.S.* no statistically significant difference, %BW^*^Ht Patient’s body-weight times body-height

### Gait analysis

At a minimum of 9 months from TKA, 3-D knee kinematic analysis was performed using a multi-camera optoelectronic system (Qualisys AB, Gothenburg, Sweden) and a force platform (Bertec Corporation, Columbus, OH) embedded in the middle of a 10-m walkway. This technology was used to analyze and compare the gait of bi-cruciate substituting TKA patients and healthy controls. Video recording and force data were synchronized and collected at 120 Hz. Gait data was collected using a Point Cluster Technique (PCT) with markers placed at reproducible anatomic landmarks on the lower limbs: nine markers were placed on the thigh and six markers on the leg to track relative motion of the lower extremity. Static trial data was collected to obtain the appropriate reference frames and inverse dynamics were used to evaluate the kinematics of the knee (angles and moments) as previously described by the current authors [1]. The BioMove software (Stanford University, Stanford, CA) was used to monitor and measure normalized knee joint moments during the stance and swing phases of gait [13]. All subjects (healthy controls and post-TKA patients) performed three walking trials at their self-selected normal pace. Kinematic data including knee flexion angle at heel strike (KFH), peak midstance knee flexion angle (MSKFA), maximum and minimum knee adduction angle (KAA), and knee rotational angle at heel-strike was extracted by the bioengineers. Peak joint moments included the first peak knee adduction moment (KAM), peak knee flexion moment (KFM), and the peak internal knee rotational moment (KIRM). Particular attention was paid to the evaluation of the external moments, using a standard inverse dynamics approach that was normalized to percent bodyweight and height (%BW%Ht) to facilitate the final comparison between participants in different groups. Final data was averaged for the different walking trials. Clinical outcomes scores (KOOS) were collected prior to the gait test to ensure that gait data were acquired in a patient population which reported a satisfactory clinical outcome. Differences between the treatment group as a whole and the control group were also assessed using student’s t-tests: significance was set at *P* < 0.05, with trends *P* < 0.15. A post-hoc power analysis was performed since the number of patients included in the study was limited: the study incidence was set at 20% in the TKA group and at 80% in the control group.

## Results

Patient demographics are presented in Table 1. There were no significant differences in BMI, sex, age, or clinical outcome score between TKA patients and healthy controls. The study population was divided into two groups: bi-cruciate substituting PS TKA and Healthy Controls.

### Knee flexion angle at heel-strike (KFH)

**Fig. 1:**
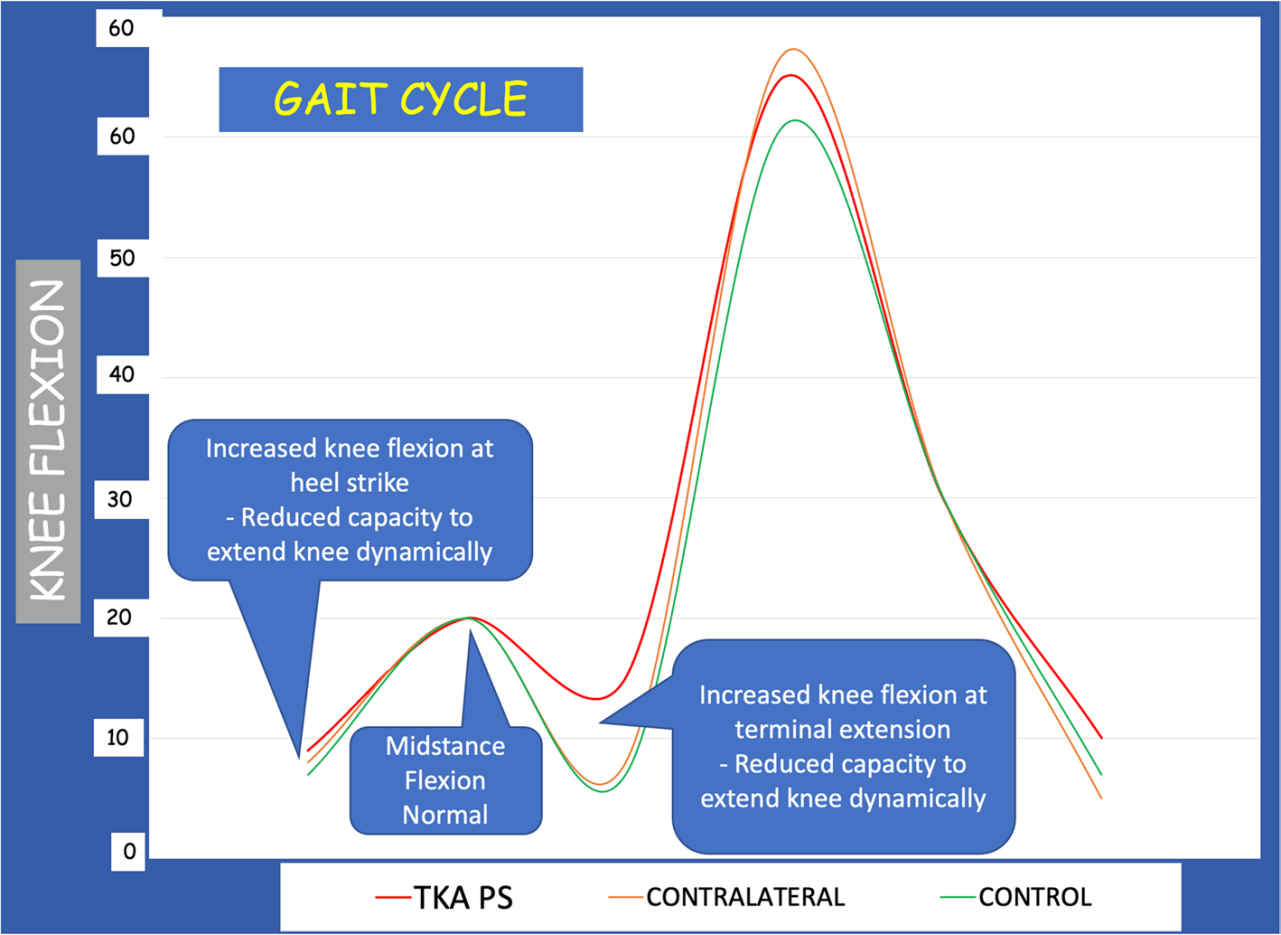
Analysis of knee flexion during Gait Cycle. A Heel-strike: both groups (PS TKA and healthy controls) showed a slight flexion contracture at heel-strike. The difference between the two groups was not statistically significant. B Mid-stance flexion angle. There was a trend that the average mid-stance knee flexion angle in the bi-cruciate substituting PS TKA group (16.1°) was lower than the healthy controls group (20.7°) (*P* = 0.07)

No statistically significant difference in KFH was observed between the two groups. The average KFH was 5° in the TKA group and 4.5° in the control group. This finding was not surprising because studies from the current authors’ Institution previously demonstrated that, with aging, the tibia undergoes a more forward inclination, and the femur has less forward inclination accordingly [13, 15]. The evaluation of this parameter showed that an increased knee flexion at heel-strike might reflect a reduced capacity to extend the knee dynamically (Fig. 1).

### Mid-stance knee flexion angle

The average mid-stance knee flexion angle in the bi-cruciate substituting PS TKA group (16.1°) trended lower than the control group (20.7°) but did not reach statistical significance (*P* = 0.07) (Table 1, Fig. 1). Interestingly, the degree of mid-stance knee flexion angle correlated with pain, as reported in the KOOS score (pain section), in both groups—patients with higher mid-stance knee flexion angles reported less pain and better functional outcomes (*P* = 0.02).

### Tibial rotation at heel-strike

The bi-cruciate substituting PS TKA group showed significantly less rotation at heel strike (5.8°) compared to healthy controls (11.2°) (*P* = 0.04). This strong difference has been related to the fact that both cruciate ligaments have been removed during the surgical procedure.

### Knee adduction angle (KAA) and Knee Adduction Moment (KAM)

**Fig. 2:**
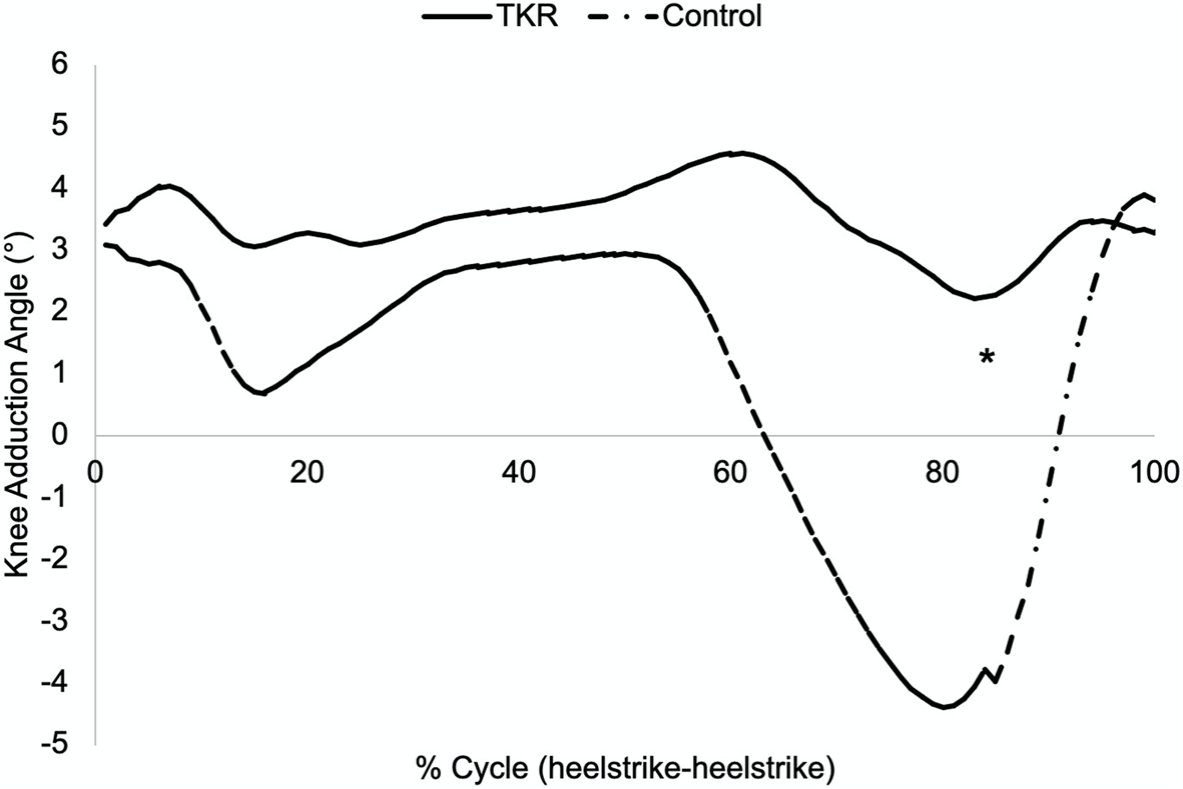
Analysis of the knee adduction angle (KAA) during the swing phase of gait. The bi-cruciate substituting PS TKA group showed less knee adduction angle excursion (peak-to-peak) compared to healthy controls

Significantly less fluctuation in knee adduction angle was seen during swing phase in the bi-cruciate substituting PS TKA group compared to the controls; the TKA group showed less peak knee adduction angle excursion (0.2°) (peak-to-peak) compared to healthy controls (-5.1°) (Fig. 2). Interestingly, the KAA excursion during swing trended higher in the healthy control group but did not reach statistical significance (*P* = 0.07).

Analysis of the Knee Adduction Moment (KAM), defined by the authors as percent bodyweight and height (%BW%Ht), did not reach statistical significance between the two groups.

### Peak Knee Flexion Moment (KFM) and Peak Knee Rotational Moment (KIRM)

**Fig. 3:**
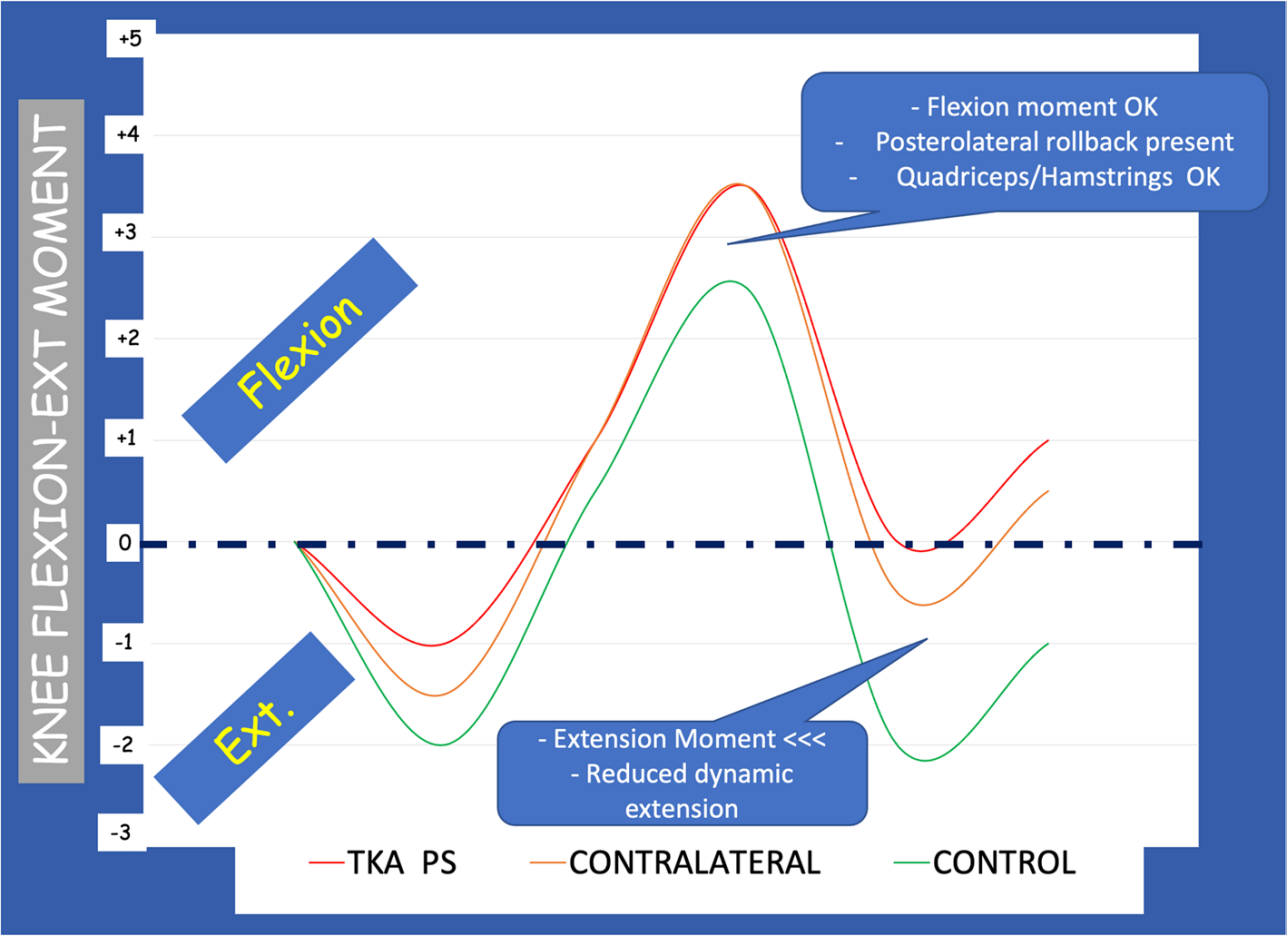
Analysis of the knee flexion–extension moments during gait. The bi-cruciate substituting PS TKA group had less late stance peak extension moments compared to healthy controls

**Fig. 4:**
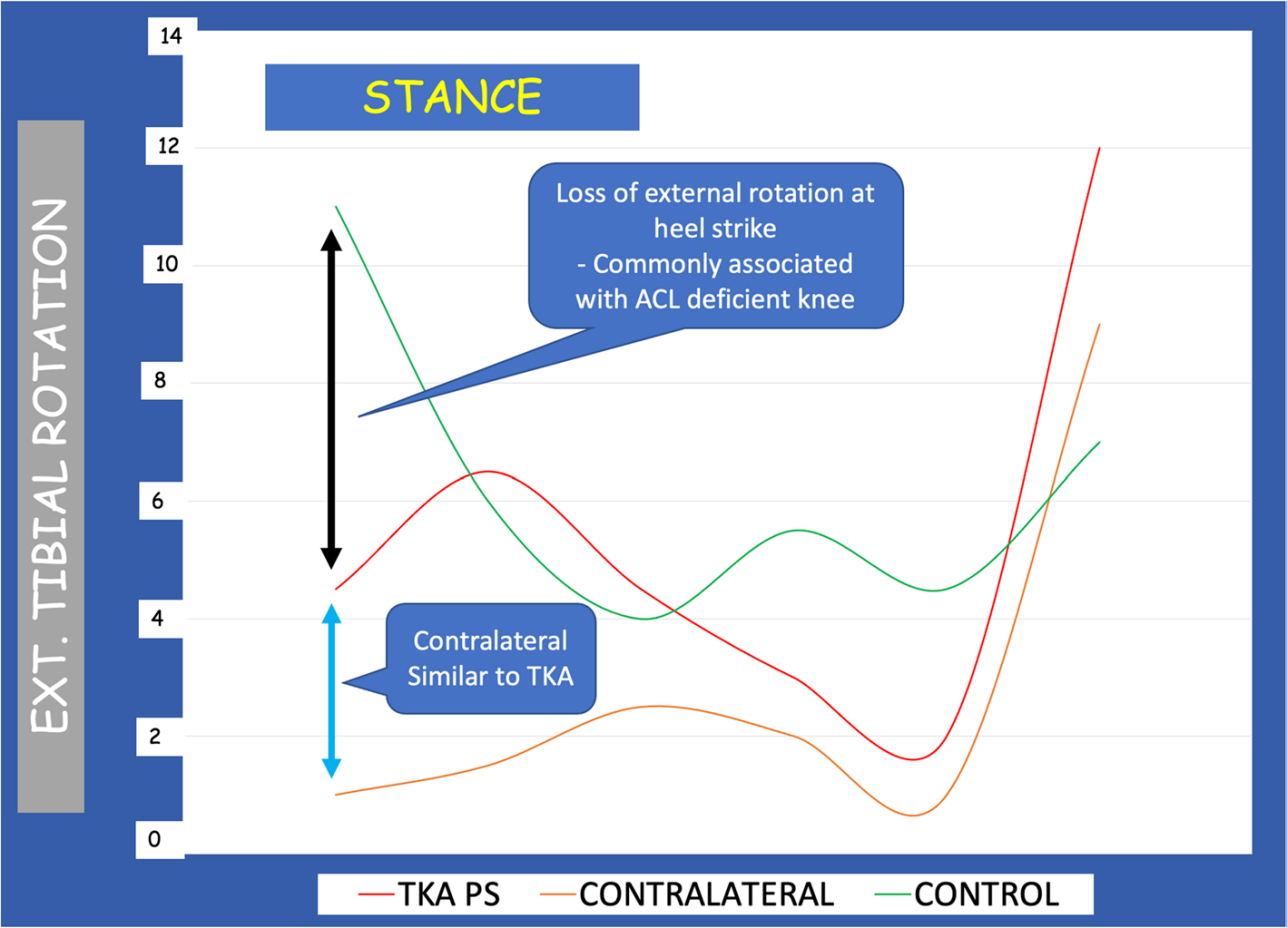
Analysis of the external tibial rotation during the stance phase of gait. The bi-cruciate substituting PS TKA group showed a significant loss of external rotation at heel strike, both in the prosthetic knee as well as in the contralateral knee. This paradox motion is typical of anterior cruciate ligament (ACL) deficient knees

The analysis of the Peak KFM did not demonstrate a statistically significant difference between the two groups (Fig. 3). Still, the bi-cruciate substituting PS TKA group showed smaller late stance peak extension moments compared to healthy controls (*P* = 0.02) (Fig. 3). No statistically significant differences were observed between the two groups in Peak KIRM. Interestingly, a statistically significant difference in the rotational moment was seen between the groups at heel strike: the bi-cruciate substituting PS TKA group had a significant loss of external rotation, a finding that is commonly associated with ACL-deficient knees (Fig. 4).

The post-hoc power analysis of the study revealed a post-hoc power of 81.7%.

## Discussion

The current study confirms that modern, third-generation, bi-cruciate substituting TKA failed to reproduce normal knee kinematics. Another major finding of this study was that the intraoperative use of a real-time sensor technology system, which has supported the surgeon in his/her intra-operative decisions, did not have a major impact on the final knee kinematic.

Despite the multiple different total knee arthroplasty designs that have been recently developed and recommended for the theoretical advantage of restoring normal knee kinematics and meeting the demands of active patients, as few as 7% of patients report that their knee feels “normal” after TKA [24, 32, 35]. While the exact cause/causes of this dissatisfaction has/have yet to be elucidated, it has been hypothesized that to improve patient satisfaction, the kinematic patterns of the implanted knee should be similar to those of a healthy knee [39]. Multiple studies have suggested that the evolution of component designs and introduction of new technologies does not always translate into restoration of native knee kinematics [12, 43].

Multiple authors have shown that the motion of the normal knee joint depends on the interaction between the shape of the articular surfaces and the ligaments crossing the knee joint [3, 39]. In the normal knee, the femoral condyles undergo a combination of rolling, sliding, and rotation on the tibial plateau during flexion. With increasing flexion, the posterior translation of the tibiofemoral contact point is greater on the lateral plateau compared to the medial plateau. This is due to the larger radius of curvature of the lateral femoral condyle. This well-established asymmetry in condylar motion during knee flexion imposes passive internal rotation of the tibia with flexion. The opposite rotational motion (“screw home” rotation) occurs when the tibia passively externally rotates during knee extension as the medial femoral condyle articular surface is wider than the lateral one [3].

Posterior stabilized knee designs have been thought to better reproduce femoral rollback and posterior translation of the femur; this is due to the engagement of the cam–post mechanism at 60° of flexion, preventing anterior femoral translation—well known as “paradoxical motion” [35]. The antero-posterior (AP) stability, normally guaranteed by the presence of an intact PCL, has been also reproduced by the use of ultra-congruent (UC) polyethylene inserts, characterized by the high conformity between the tibial and femoral articulating surfaces [4, 25].

In the current study, no statistically significant differences were found between the two groups in peak KFM during level walking. Interestingly, patients with designs that remove or substitute for the PCL tend to reduce their knee flexion moment and thus the resulting demand on the quadriceps. In PCL substituting TKAs, the lack of femoral rollback reduces the lever of the quadriceps and thus its mechanical efficiency. This reduction usually manifests at about 60° of knee flexion, the angle at which the greatest demands are placed on the quadriceps during stairclimbing. Consequently, the mechanism that patients use for the adaptation is a forward lean of the torso [3–5].

In this study we also observed a significant loss of external rotation at heel strike and a trend toward a lower average mid-stance knee flexion angle in the PS group compared to the healthy controls. The current authors strongly believe this finding could be related to the absence of the ACL, which contributes to the external orientation of the tibia at full extension (“screw-home” mechanism in the normal knee). The role of the ACL is also to limit the anterior displacement of the tibia with respect to the femur in early to mid-flexion [39]. It has been established that ACL deficiency can cause the avoidance of quadriceps contraction during activities when the knee is near full extension. It appears that patients adapt to the absence of the ACL by minimizing the demand for quadriceps activation as the anterior pull of the patellar ligament is no longer stabilized by the ACL. Andriacchi et al. confirmed that patients with an ACL-deficient knee had a significantly lower than normal net quadriceps moment during the mid-portion of the stance phase of walking ("quadriceps avoidance" gait). In the normal knee, the quadriceps muscles control knee flexion to approximately 20° during midstance; the patients who underwent TKA in the current study did not flex the knee in the same manner [4, 6, 11]. In ACL deficient knees, quadriceps muscle strength deficit contributes to reduction of the knee angles and moments as a natural reaction to knee instability [19, 29]. These historical findings matched our results, which demonstrated a trend toward a lower mid-stance knee flexion angle in the PS TKA group (16.1°) as compared to the healthy control group (20.7°). Moreover, in our series, lower mid-stance knee flexion angle correlated with worse KOOS pain scores.

The recent use of polyethylene designs alternative to the classic PS designs, especially medially constrained, has recently increased with the hope of reproducing normal knee kinematics and to mimic the physiologic medial pivoting pattern with posterolateral femoral roll-back in flexion [14], This “kinematic advantage” was hoped to translate into improved knee proprioception by the patient. Despite successful clinical outcomes, greater patient satisfaction, and higher forgotten joint scores (FJSs) when compared to traditional PS designs, the kinematic patterns of these designs failed to be similar to those of normal knees [16, 18, 23, 37, 41].

In the current, the use of sensor-embedded tibial inserts did not reproduce a kinematic pattern similar to the normal knee during standard gait analysis: the authors’ interpretation of this finding was that the retention of the cruciate ligaments along with a reproduction of the articular geometry, could produce a more normal knee kinematics, greater stability, and decrease muscle forces leading to improved knee function. In a previous study, Meneghini et al. [30] reported higher satisfaction and better Knee Society Function scores when early flexion lateral pivot and late flexion medial pivot kinematic patterns were detected intraoperatively by sensor-embedded tibial inserts [30]: in our study, unfortunately, a correlation between the acquired kinematic data and the clinical results could not be done due to the small cohort analyzed, representing a major limitation of this study.

Bicruciate-retaining (BCR) TKA designs have been developed in the past to reproduce knee biomechanics through ACL preservation: the current literature on the use of BCR TKA designs is extremely controversial: a strong debate is still ongoing between supporters [21, 26, 36, 45] and opponents [7, 8, 19, 36, 39].

Few authors [9, 17] have recently reported excellent outcomes and near- normal gait characteristics when patients who received Bi-unicondylar arthroplasties (Bi-UKA) were matched with TKA subjects: the current authors agree that this technique could represent an interesting surgical strategy in modern total knee arthroplasty.

The main limitation of this study is the small cohort of patients which precludes from making a correlation between the kinematical analysis and the clinical results; however, considering that existing kinematic studies traditionally have relatively small numbers, the authors believe this work could provide useful information for future studies on kinematics following knee replacement. The post-hoc power of the study was 81.7%.

The authors also recognized that, in the gait analysis section of this study, more activities should be analyzed in addition to walking on level-ground: this will eventually help to get a better understanding of the actual knee kinematics.

## Conclusion

Modern, bi-cruciate substituting TKA designs failed to reproduce normal knee kinematics despite the intra-operative use of sensor-embedded tibial inserts. The loss of rotation during heel strike, the lack of the “screw-home mechanism,” and the lower mid-stance knee flexion angle all represent major proprioceptive and biomechanical differences between normal and replaced knees. Implants that preserve both cruciate ligaments and allow the reproduction of normal articular geometry in association with robotic-assisted personalized alignment should be studied to detect how far we are from reproducing a closer-to-normal knee kinematics.

## Data Availability

Data are available upon request to the corresponding author.

## Abbreviations

TKA: Total knee arthroplasty
MC: Medially-congruent
PS: Posterior-stabilized
KFH: Knee flexion angle at heel-strike
MSKFA: Peak midstance knee flexion angle
KAA: Knee adduction angle
KAM: Knee adduction moment
KFK: Peak knee flexion moment
KRM: Knee rotational moment
